# Clinical stance on response initiation in autistic adults: co-creating an integrative approach based on theory and lived experiences to act from language, via motor movement to wellbeing

**DOI:** 10.3389/fpsyg.2023.1229596

**Published:** 2023-09-14

**Authors:** Kirstin Greaves-Lord, Manna Alma, Benjamin de Graaff, Jeanet Landsman, Klaske van der Weide, Gabrine Jagersma, Theo Beskers, Marieke Wubs, Henri Mandemaker, Emma van Daalen, Joost van der Linde, Anne Fleur Stapert, Jeroen Bekius, Sigrid Piening, Annemiek Landlust, Ingrid D. C. van Balkom

**Affiliations:** ^1^Jonx, Department of (Youth) Mental Health and Autism, Autism Team Northern-Netherlands, Lentis Psychiatric Institute, Groningen, Netherlands; ^2^Department of Psychology, Clinical Psychology and Experimental Psychopathology Unit, University of Groningen, Groningen, Netherlands; ^3^Department of Health Sciences, Applied Health Research, University of Groningen, University Medical Center Groningen, Groningen, Netherlands; ^4^Mental Health Institution GGZ Rivierduinen, Leiden, Netherlands; ^5^PAS Nederland, Hague, Netherlands; ^6^Vrij Leven ACT Coaching, Dordrecht, Netherlands; ^7^Nederlandse Vereniging voor Autisme, De Bilt, Netherlands; ^8^Department of Child and Adolescent Psychiatry/Psychology, Erasmus MC, Sophia’s Children’s Hospital, Rotterdam, Netherlands; ^9^Department of Psychiatry, Rob Giel Research Centre, University Medical Center Groningen, Groningen, Netherlands; ^10^Department of Genetics, University of Groningen, University Medical Centre Groningen, Groningen, Netherlands

**Keywords:** immobility, autism, inertia, activation, inclusive society/spaces

## Abstract

Getting ‘stuck’, literally and figuratively, is a common experience for autistic people. Literally ‘stuck’ means exhibiting limited response initiation due to immobility with tense muscles and inability to move. Figuratively ‘stuck’ means loneliness, passivity or captivity in activities that do not offer long-term satisfaction. To further conceptualize this complex phenomenon of limited response initiation in autistic individuals, we performed qualitative interviews and focus groups with autistic people and their family members, followed by brainstorm sessions and a Delphi study with input from a larger panel of experts from multiple backgrounds. We aimed to co-create the outline of an integrative approach to support autistic people in moving away from this ‘stuck state’ to more flexible, limber ‘supple states’ in order to live freer, more meaningful, satisfying and peaceful lives. Over time, in interaction with all participants, our shared insight grew. Based on this, we here stipulate a conceptual framework, in which the described ‘stuck state’ at the micro-level of the muscles/behavior of one individual, probably is caused by feeling/being ‘stuck’ or ‘cramped’ at several overarching (i.e., meso and macro) levels. For instance, stuck in relationships with unhealthy dynamics, stuck at home creating short-term calm, trance-like states (e.g., gaming), stuck at an educational level that might fit the individuals’ current social–emotional state rather than their potential cognitive level, stuck in a job that pays the bills but does not feel meaningful, nor contributes to a satisfying life with opportunities for personal development. Stuck in a mental/public health care system where ever ongoing changes in policies hinder sustained support to suit care-needs. Stuck in a regulated societal system making it likely to repeatedly get stuck. Is this phenomenon specific to autism? Formally we have only conducted interviews with this population, but in another smaller, related project we also spoke to people from the general population with careers that are considered successful in the general society. These people actually voiced similar experiences. Therefore, we hypothesize that this numbing state of being or feeling ‘stuck’ may be a prevalent phenomenon that needs to be addressed. In this article, we discuss several types of interventive approaches (i.e., language-based talking therapies, affective experiential expressive therapies, physical therapies and systemic therapies), prevention as well as intervention programs, directed at different primary stakeholders, that can complement and enrich each other in an integrative policy, that leads to tailor-made, personalized trajectories of interdisciplinary support to enable people to live satisfying, meaningful, dignified and peaceful lives.

## Introduction

Getting ‘stuck’, literally and figuratively, is a common experience for many autistic individuals*. With literally ‘stuck’ we mean immobile with cramped, tense muscles, a limited ability to move or initiate a response, in the most severe cases catatonia (Dell’Osso et al., 2022). With figuratively ‘stuck’ we mean experiencing feeling stuck, for instance a limited/loss of ability to motivate oneself to action, physically and/or mentally, and/or limited ability to get moved (emotionally), get out of an unwanted emotional state, or get motivated by others, feeling stuck, cramped or captivated in oneself (loneliness), showing passivity or feeling stuck in activities that do not (no longer) offer long-term satisfaction (Greaves-Lord et al., 2022).

This ‘state of stuckness’ was previously also referred to as ‘autistic inertia’ (Buckle et al., 2021). Investigating factors contributing to ‘behavioral problems’ in autistic people in a qualitative study on lived experiences raised the topic and emphasized the importance of the lack of response initiation (i.e., the Grasp on Behavior project, Greaves-Lord et al., 2022). In short, dropping out of school, work and/or other activities while communicating (either verbally/non-verbally) their reduced motivation to participate, led to their behavior being considered by others as problematic/oppositional. However most of our participants explained that this behavior was not intended to cause problems, but originated from a ‘stuck state’, i.e., limited response initiation. In a second project (i.e., Dealing with one’s own Autism) we gained more knowledge of this state of ‘stuckness’ when performing in-depth interviews with autistic adults.

In the Methods and results sections of this paper, we will introduce and subsequently present the findings of both projects that led to a conceptual outline of an integrative approach, i.e., an approach in which a customized mix of guidance and therapy is made—and decided upon in equally shared decision making—over the life course and at intersections between life domains, existing of an integration of complementary components. This conceptual outline and concrete suggestions regarding components from existing methods/services will be presented in the Discussion.

The ultimate aim of the integrative approach is to enhance more flexible, limber ‘supple states’ in order to create freer, more meaningful, satisfying, dignified and peaceful lives.

* throughout this article, we will use the terms autistic people, people with autism, or autism spectrum disorder in an interchanging way, as we hope to attune to the needs and desires of all involved and do not wish to insult anyone with the use of our wordings.

## Methods

This perspective paper results from the Dutch Academic Workplace Autism (AWA; https://www.autisme.nl/over-autisme/onderzoek-naar-autisme/academische-werkplaats-autisme-awa/). The AWA was founded in 2013 by Prof. dr. Hilde Geurts and Dr. Kirstin Greaves-Lord. The AWA program was commissioned by the Dutch Ministry of Public Health, Wellbeing and Sports, in response to a report of the Health Council of the Netherlands. Autism spectrum disorders: a lifetime of difference (The Hague Health Council of the Netherlands, 2009; publication no. 2009/09). This report signaled the fragmentation in the Dutch system (i.e., the organization of healthcare, well-fare, educational services, etc.) as a presumed cause for the unmet care needs of autistic people. Funding the AWA on the short term allowed for improved intersectional collaboration through the implementation of a knowledge infrastructure, while on the longer term aimed to better meet the needs of autistic people and their families. Once the foundational collaborative connections were established, in 2016 additional funding was assigned for a larger collaborative project [i.e., Grasp on Behavior (GoB)] focusing on youngsters who were entering into adulthood and who had traits/a classification of autism spectrum disorder as well as oppositional behavioral disorder, and who were not/no longer participating in education and/or occupation. The goal was to identify factors contributing to the limited societal participation of this population. This participative project entailed four components: (a) a literature study to gain an oversight of the empirical information on the topic so far, (b) focus groups with all relevant stakeholders involved to obtain their reflections on the outcomes of the literature study, (c) interviews with autistic individuals and their proxies, and (d) a Delphi study to make an inventory of potential products/services considered helpful by most stakeholders, i.e., meeting the needs of autistic people and their loved ones to overcome any obstacles in their societal participation. This process was conducted by an interdisciplinary AWA program group consisting of people with autism, proxies, professionals from science, care, education, experience and/or policy. As part of component c (the interview study) thematic analyses were performed, using both a deductive and inductive approaches (Braun and Clarke, 2006).

The outcomes of the GoB project were subsequently used in a follow-up project called ‘Dealing with (one’s own) Autism’(DwooA). This project started in 2019. Besides building on the results of GoB, DwooA also was founded based on the results of the AWA Knowledge Agenda project. This AWA-broad inventory amongst 1,169 stakeholders further clarified needs to overcome gaps between science and practices (Van den Bosch and Greaves-Lord, 2019). The DwooA project substantiated the insights from the GoB and Knowledge Agenda projects by (a) performing in-depth individual interviews with autistic adults regarding specifically identified themes, (b) further discussing the outcomes of these interviews in focus groups with autistic adults and with their near ones, (c) a second Delphi study to more concretely clarify and organize the goals of the products/services that should be developed/optimized, and (d) an inventory and analyses of existing methods/services deemed useful by the members of the interdisciplinary AWA program group. Again, within this participative study process, thematic analyses were performed, using both a deductive and inductive approaches (Braun and Clarke, 2006). In all stages, to optimize trustworthiness, we followed the recommended procedures from the literature (i.e., Guba, 1981; Sandelowski, 1993), such as coding with two researchers and subsequently performing peer debriefing and member checks, to make equally shared decisions theme/goal names etc. In both projects, we used maximum variation sampling (Coyne, 1997), and an iterative approach was adopted in which themes were constantly finetuned amongst the program group members.

In the following section, we will summarize the results from the GoB and DWooA projects that accumulated towards the conceptual outline of the integrative approach.

## Results

### GoB

Since varying factors were identified that contributed to the limited societal participation of participants, we organized these factors according to their level in the ecological systems of human development (Bronfenbrenner, 1979), i.e., at the micro, meso or macro level of society:

#### Micro-level

##### Self-insight and (self-)acceptance

Most participants reported a lengthy diagnostic process to reach a first diagnostic classification of Autism Spectrum Disorder (ASD; i.e., according to the DSM 5) and most struggled to accept the diagnosis:

‘I did not like one bit of it… I am still not really accepting it [the diagnostic classification ASD]. I just totally do not feel autistic’ (A1♀).

‘I thought “No, that cannot be, that is not what I have, that’s the children I see at my mom’s work, you know? That is not what I have.” I thought; “Yeah sure, big bye bye to you!” (A2 ♀).

Usually a late diagnosis of ASD was preceded by other classifications that did not fit as the participant’s insight in own functioning increased in treatment (e.g., due to psycho-education).

##### The way others approach you

Some participants felt under-estimated by others:

‘“She could go to a sheltered workplace or school, but more should not be expected”, was said about me, but now I have completed intermediate occupational education and have work experience’ (A3 ♀).

The opposite was also true, adolescents with ASD could also get over-estimated when based on cognitive functioning (verbal/performance intelligence) a certain educational level seemed feasible. However, slower processing speed or atypical social–emotional maturation, may have hindered academic achievement and as a result, these teenagers may have constantly overextened themselves, building up tension and frustration over time:

‘First with me it was obvious, I had high grades and my homework done in no time, so yeah, it was obvious I would do pre-university education. Yet later, things became too much, I began getting failing grades proving I had actually been walking on tiptoes without anyone noticing it’ (A4 ♀).

#### Meso-level

##### Education

Participants reported considering the massiveness of and chaos within schools to be detrimental. They felt it was crucial to experience overview and structure:

‘Then the bell would ring, everybody packing bags and leaving, with the teacher still calling “o yeah, the assignment for next week is this and that” (A5 ♀).

The need for structure might be obvious to people familiar with autism. And although the exact embodiment of this need might differ from person to person, it seems quite fundamental to most people on the spectrum. Yet, one of our participants insisted very strongly that while we might think this need for structure is a ‘no-brainer’, in fact this was not the case for some educational experts, with some teachers not knowing or not acting accordingly.

Other participants mentioned difficulties in transferring from special primary education to main stream secondary education, because of the lack of adjustments for sensory differences. Participants indicated the sensory overload experienced made it difficult to keep an overview of activities, tasks, and adhere to a realistic time schedule. As such, offering a realistic schedule and support to adhere to it, is essential to prevent sensory, cognitive and/or affective overload.

Although one participant also emphasized that school policy measures should not be exaggerated, it was important to create calm areas to regulate senses and tension, but certainly as important to offer a suitable (cognitively) stimulating environment to train both knowledge and skills for a world rich in constant stimuli.

Finally, one of our program group members mentioned that he especially observed drop-outs during internship periods. This might be due to the fact that the employees coordinating these internship usually are not informed about the diagnosis of pupils, and as such, the organization that offers the internship is also not informed. Finding a suitable moment and person to open up to about relevant vulnerabilities is therefore an important yet delicate issue.

##### Community support: social work/coaching

Most participants report finding positions at their current school/job, due to the support of a school-support worker/ coach/intermediate social worker, as this support helped find the most suitable fit for their needs and desires:

‘My coach helped me in a way that matched me; not pushing me constantly, that is what works for me’ (A6 ♀).

‘My son now has a clear go-to-person. He gets overwhelmed easily and then starts showing annoying behavior. If he can immediately direct his question to the designated person, then he avoids getting overwhelmed’ (P1 ♀).

‘I have a weekly meeting with my coach to help me keep a tight agenda. I now oversee what I have to do and how much time that will take me’ (A7♂).

‘A social worker, quite a feisty one, who was clear about boundaries and a good kick in the butt. Without her I would not have graduated’ (A8♂).

#### Macro-level

##### Separate funding streams

In the Netherlands, organizing appropriate services over time is complicated, as funding for psychological or social support has different sources. Access to mental health care requires a diagnostic classification while social services policies differ amongst municipalities:

‘As a support worker, I got to know which consultant to talk to at which council to get the right amount of support within a certain school for my pupils. Yet, it has been very frustrating, since for my own child, by no means I can get such support, as at this school, they work with a designated contractor that only has one hour a week per pupil. This makes me think, how bizarre!’ (P2 ♀).

Participants emphasize that not only support for the autistic person might be needed, but also for their proxies to support them. Moreover, support should not be removed as soon as the situation gets better, as this improvement is probably due to the support being there.

##### Informal network

Besides formal support, the proximity of informal social support is enabling participation in school/work. Such relations support in making decisions and having their back when the going gets tough:

‘My mom can keep her patience very well. When all the anger blew over, she was a good support. Content-wise I could not always turn to my friends, but I could relax with them, which is important in its own way’ (A9♂).

Especially a sense of belonging is important and might explain why some behaviors are considered anti-social:

‘At one time, this guy kept following my friend, which really freaked her out. So I went up to him and told him to leave her alone. We ended up in a big physical fight… which did put me in trouble… but I don’t mind, ‘cause he was harassing her, and some people might consider my friends weirdos, but they are mý weirdos, you know, no matter what!’ (A10♂).

Parents often are the mediator between school, health care and their child. Some parents did back out for a while, but when not interfering, their child’s situation escalated. For instance, one parent had to pick up their child from the street, because her son had turned homeless. Obviously, this takes much energy:

‘To stay on top of it, not letting go, is needed, ‘cause if I won’t, we will all collapse. Our kids should feel they can rely on their parents, cause if they can’t, what’s left?’ (P3 ♀).

##### Collaborative integration

Many participants indicate that collaborations between schools, care, social support, parents and youngsters often fail. Plans are made, but not persevered. Communication is often hampered or poorly timed.

Interrelations between the factors described above get over-looked very often. For instance, externalizing problems (i.e., anger, aggression at the micro-level) are actually fueled by internalizing problems (i.e., worry about what might go wrong at the meso-macro-level). Managing problems at the meso-/macro-level (i.e., school drop-out, declined societal participation) can only adequately be done, when timely signaling problems at the micro-level (i.e., sensory, cognitive, and/or affective overload that are brewing the tension). These signaling skills, can only evolve over years and need to be developed and matured, fed by lived experiences, introspection and reflection. Such reflection can be facilitated in self-conceptualization, psycho-education and emotion regulation interventions. Yet, often structured booster sessions over time will be needed to keep bolstering the learning process, and help autistic people ‘to connect the dots’ within their often over-loaded minds that are stored with many detailed and affectively-charged memories.

### Results DWooA project

In the subsequent DwooA project we further delineated the phenomenon of stuckness. In the interview-schedule, we departed from potential problems at the micro level, but findings also reflected phenomena at the meso and macro levels (please see depicted in Supplementary Figure 1). More in-depth results and quotes from these interviews can be found in Table 1.

**Table 1:** Summary of DwooA results.

Themes	Explanations of processes	Example quotes
1. Getting to know one’s self – constructing a suitable conceptualization of the self over time (~Knowledge)	a. When participants do not know their selves, this on the short term leads to constant doubt and worries, and on the long run leads to insecurity, identity confusion and exhaustion b. One obstacle in getting to know the self, is the physical recognition, and subsequent naming and interpreting of own emotions c. Another obstacle in getting to now one’s self is the difficulty with social situations. Although participants recognize that social contacts are essential to get to know one’s self, these are often avoided due to the awkwardness they evoke.	a. ‘What happens around me, has a huge impact on me. If I do not even know how I work, it gets a total mess. I mean total confusion, that you do not know what’s going on from head to toes, who you are, what is happening, just not having solid ground’ b. ‘I only became aware of my boundaries later in life, when I had crossed my physical and emotional boundaries seriously, resulting in burn-out and no longer being able to meet obligations’ c. ‘When going to main stream secondary education, I got confronted with social things going faster, people taking less consideration or account of you, groups being more massive. This was a huge mountain… like fall and rise again, that’s how it goes, but eventually this is a very important way to learn’
2. Staying true to the self (the necessities and requirements for fitting in) (~Acceptance)	a. Afraid for not being accepted, emotions are often only shared once they have accumulated over time, which may lead to conflict. b. Several strategies are used to come across socially acceptable, deliberately or less consciously, like camouflaging (hiding certain aspects of the self), acting (taking on a role that does not suit genuine feelings/needs), avoidance (i.e., of social situations due to a fear of rejection, while a desire for true connection is felt) and imitation (mimicking others behaviors). Using these techniques feels like lying, takes a lot of energy and estranging from the self and others, resulting in depression. c. To stay true to one’s self, knowing and expressing your boundaries is necessary, and to have faith that other people will not reject or judge you.	a. ‘If it really gets too much, then it becomes hard, because then eventually I do share but it comes out real shitty with loads of emotion, which really startles my husband’ b. ‘There were periods of depression that arose from the role that I took on getting so big, strong and present that it took over my entire life. I would look around me, beautiful career, accomplished, but it felt like someone else’s career, that of my persona, my self was no longer present’. c. Practicing faith could be something to do more. To trust people, that they will not reject or judge you, without a clear reason, trusting that people are actually quite accepting, without constant judging’
3. Creating and maintaining a sense of belonging (the ongoing search for meaning in life) (~Regulation)	a. When for instance a job is lost, or cherished dreams for the future cannot be realized, lack of meaning can be experienced. When there is nothing to live forward to, insecurity, boredom and mental health issues arise, like existential fears and frustrations, numbness and suicidal ideation. b. Participants mention the desire for a place and activities in which their qualities can be expressed, where there is room for vulnerability and where you can add meaning. The thought that it is not always about the result, but about the effort and dedication provides rest.	a. ‘What am I doing it for? What can I add? Looking for answers I will not get, which gives restlessness, insecurity, resulting in depression, a downward spiral. There should be some purpose to life? And that sure of hell is not sending bills!’ b. ‘Time pressure is a killer for my brain!’. ‘I’d like less social pressure, where the priorities of the social surroundings are placed above my own priorities’.
4. Finding and remaining one’s position (the challenges of authentically presenting the self in social settings) (~Positioning)	a. Many participants were rejected/bullied in the past, or felt like not fitting in. Clarity and predictability are important prerequisites to feel save in a certain position. b. To feel safe in a position, honesty and being accepted are other important prerequisites	a. ‘We had this new intern who was very blunt, like ‘poor the coffee’. But when it comes to me, this is so clumsy, cause I’m like ‘which can, which cups, where, on a plate? And why? ‘I need more details and context!’ b. ‘To feel like, here I can say everything, I do not have to be careful to make weird mistakes, say ‘autistic things’. The fact that you can behave in your own way is such a relieve!’

## Discussion

The Academic Workplace Autism set out to better understand the limited societal participation of autistic young adults. The GoB project showed that much of the behaviors that were interpreted by others as intentional refusal to participate, or active avoidance of activities, could in fact be better understood as ‘autistic inertia’ (Buckle et al., 2021), i.e., a lack (limited capacity) to initiate activities, not being able to activate one’s self to come to action, being ‘stuck’. This ‘stuck state’ can be described at micro-level as muscles/brain literally being/getting stuck/cramped. Yet, very interestingly, GoB participants indicated that this state is probably caused by more figuratively feeling/being ‘stuck’ or ‘cramped’ through several overarching and accumulating (i.e., meso and macro) levels. For instance, stuck in relationships with unhealthy dynamics, stuck at home creating short-term calm, trance-like states (e.g., gaming), stuck at an educational level that might fit the individuals’ current social–emotional state rather than their potential cognitive level, stuck in a job that pays the bills but does not feel meaningful, nor contributes to a satisfying life with opportunities for personal development. Stuck in a mental/public health care system where ongoing changes in policies hinder sustained support to suit care-needs. Stuck in a regulated societal system making it likely to repeatedly get stuck. These varying concrete expressions in reality of this ‘stuck state’ at the different levels of society (micro, meso, and macro) are depicted in Supplementary Figure 1.

Is this phenomenon entirely specific to autism? We do not think so. We also participated in the Self Coach App (SCA) project. This project covered similar (existential) themes in an interview study, but targeted people from the general population who successfully participated in higher education or higher income occupations, yet who struggled with psychological and existential issues. These people actually voiced similar experiences, such as avoiding activities, feeling pressure to succeed, and not always knowing who to turn to when in sorrow. Yet, our clinical experience with autistic people who got severely stuck, learned us that potentially the combination of heightened arousal and related stress at several social domains, limited self-regulation and reduced *in-vivo* social problem solving skills might put this group of people at a higher risk to get and maintain to stay stuck than allistic people with mental health issues.

Therefore, we observe that this numbing state of being or feeling ‘stuck’ may be a prevalent phenomenon, that is important to resolve in order to contribute to increased participation/inclusion and meaning of life at the individual and societal level. Therefore, in the DwooA project, besides the interview study, we also conducted a Delphi study in order to specify goals on how to approach this complex issue. We reached consensus on four main goals: gaining 1. (self) knowledge, 2. (self) acceptance and/or 3. (self- and co-) regulation in order to 4. establish and maintain a suitable position in society (i.e., self-realization/positioning). The themes from the GoB + DwooA interviews and Delphi studies are depicted in an integrative thematic map, please see Supplementary Figure 1. To provide actionable recommendations on how the proposed goals could be targeted, we subsequently asked the program group their suggestions on potentially useful methods/services, i.e., interventions that they had found useful during their own personal development coping with periods of ‘stuckness’ or through observing effects of interventions in others. This resulted in the interventions inventory presented in Table 2. The inventory shows several types of practical approaches, ranging from language-based talking therapies, affective experiential expressive therapies and physical therapies to more broad-scale social systemic interventions. Interestingly, along the way, we did not just identify interventions primarily targeting autistic people themselves, but as time went by, more intervention programs were identified or developed that were directed at other primary stakeholders, such as parents, care-providers or the general public. As such, programs targeting the autistic person mainly had the purpose of intervention (i.e., increasing wellbeing/mental health), while other programs targeting the surrounding social system mainly had preventive purposes. When mapping the intervention programs to the four main goals, we realized that neither program in itself can serve all goals, especially when considered from a life scope perspective. Rather, these programs can complement and enrich each other across (and at the intersections of!) life domains and life stages. Therefore, we suggest that an integrative policy should be co-led, in which a life coach or coordinating therapist—in mutual equality together with the person with autism, proxies and professionals involved – decide on a tailor-made, personalized trajectory of interdisciplinary support and care. Such an integrative approach, in which all stakeholders involved reach consensus on which realistic goal should be prioritized at what time, which intervention suits the person at that life stage, should enable the autistic person to – in co-creation with their social support system – achieve, step-by-step with ups and downs, sufficient mental health and wellbeing and live a satisfying, meaningful, dignified and peaceful live. This approach might especially be important for autistic people, as difficulties with Central Coherence (CC; e.g., López et al., 2008), executive function (Demetriou et al., 2018) and high levels of stress (van der Linden et al., 2021) inherent to autism, might interfere with overseeing this bigger picture. Since most people in periods of increased stress/pressure, have difficulty with maintaining a birds eye view, a coach/mentor who facilitates an integrative perspective on all life domains and particular needs in certain life stages, might be a prerequisite in response initiation, in being able to benefit from services/interventions and in enhancing personal growth, mental health and wellbeing throughout life in motion.

**Table 2:** Results interventions inventory.

Level	Stakeholder	Intervention	Needs				Stage of life	IQ	Setting	Duration	Ref.
			Knowledge	Acceptance	Regulation	Positioning					
Self	Client	Acceptance & Commitment Therapy (ACT)	+	+	~	+	≥ School age	All	Mental health care	3 - 12 Sessions	(Hahs et al., 2019)
	Client	Body Awareness Therapy (BAT)	+	+	–	+	≥ Adolescence	≥ Well below average	Physical health care	10 Weeks	(Vancampfort et al., 2022)
	Client	Cognitive Behavioural Therapy (CBT)	+	~	~	~	≥ Early childhood	≥ Average	Mental health care	3 - 12 Months	(Hesselmark et al., 2014)
	Client	Competitive Memory Training (COMET)	+	+	+	~	≥ School age	≥ Well below average	Mental health care	6 - 9 Sessions	(Stella et al., 2022)
	Client	Dialectic Behavioural Therapy (DBT) in ASD with Suicidality & Self-destruction (DIASS)	+	~	+	+	≥ School age	≥ Average	Mental health care	6 - 12 Months	(Hartmann et al., 2012)
	Client	Dynamic Interactive Social Cognition Training in Virtual Reality (DiSCoVR)	–	~	+	+	All	≥ Well below average	Mental health care	16 Sessions	(Van Pelt et al., 2022)
	Client	Emotional Awareness & Skills Enhancement (EASE)	~	+	+	+	≥ Adolescence	≥ Low average	Mental health care	16 Sessions	(Mazefsky et al., 2021)
	Client	Eye Movement Desensitization & Reprocessing (EMDR)	+	+	+	+	All	All	Mental health care	2 - 6 Sessions	(Lobregt-van Buuren et al., 2019)
	Client	Find your own way with your autism	+	+	+	+	Adolescence	≥ Well below average	Self-help	Variable	(Agterberg-Rouwhorst, 2022)
	Client	Psycho-education for smart teens with ASD	+	~	~	~	Adolescence	High average	Special education	10 - 15 Sessions	(Van der Meijden & van der Stegen, 2009)
	Client	Tackling Teenage Training	+	+	+	+	Adolescence	≥ Low average	Mental health care	12 - 18 Sessions	(Visser et al., 2012)
	Client	I am special	~	~	~	~	School age - Adolescence	≥ Average	Special education	Variable	(Vermeulen, 2005)
	Client	Imagery Rescripting (IR)	+	+	+	~	Adulthood	≥ Low average	Mental health care	10 Sessions	(https://www.autismedigitaal.nl/avn/imaginaire-rescripting/, n.d.)
	Client	Less stress, less autism: Plan C	+	+	+	+	Adolescence	≥ Well below average	Self-help	Variable	(Kuipers-Hemken & Buma, 2020)
	Client	Meaning of Life Therapy	~	+	+	+	Adulthood	≥ Well below average	Mental health care	8 Sessions	(Breitbart & Poppito, 2014)
	Client	Mentalization-based Therapy (MBT)	+	+	+	+	≥ Early childhood	≥ Well below average	Mental health care	12 Sessions	(Kaltenegger et al., 2020)
	Client	My self portrait	+	+	+	~	Adolescence	≥ Well below average	Self-help	10 Sessions	(Buys & Aerts, 2011)
	Client	Psychomotor Therapy (PMT)	~	–	~	–	All	All	Mental health care	Not specified	(Frazão et al., 2023)
	Client	Re-Attach	+	+	+	+	All	All	Coaching	3 Months	(Bartholomeus, 2021)
	Client, peers, caregivers	Regulation Organization Autonomy Didactics (ROAD)	+	+	+	+	Adolescence	≥ Well below average	Mental health care, secondary education	14 Sessions	(Idris et al., 2022)
	Client	Safe & Sound Protocol (SSP)	–	~	+	-	All	All	Mental Health care	5 Sessions	(Porges et al., 2014)
	Client	Schema Therapy (ST)	+	~	–	+	≥ School age	≥ Well below average	Mental health care	4 - 24 Months	(Vuijk et al., 2023)
	Client	Sensorimotor Psychotherapy (SP)	+	~	+	-	Adults	Average to above	Mental health care	Variable	(Classen et al., 2021)
	Client	Somatic Experiencing (SE)	+	~	+	-	Adults	All	Mental health care	Variable	(Brom et al., 2017)
	Client	The full heads book	~	+	+	~	School age - Young adulthood	All	Self-help	Variable	(Kraijenhoff, 2016)
	Client	This is me!	+	~	~	~	Adulthood	All	Mental health care	Variable	(Van der Meer et al., 2021)
	Client	Virtual Reality Treatment for Social Activities & Participation (VR-SOAP)	–	~	+	+	Young Adulthood - Adulthood	≥ Well below average	Mental health care	14 Sessions	(Meins et al., 2023)
	Client	VRelax	~	+	+	–	All	All	Mental health care	14 Sessions	(Veling et al., 2021)
	Client	Yin yoga	–	+	~	–	All	All	Social domain	Variable	(Semple, 2019)
	Client	Yucel method	–	~	~	+	All	All	Coaching	Variable	(Van Wamel & Planije, 2018)
Micro	Client, professionals	ASD Guide	+	~	~	+	All	All	Social domain	10 Months	(Van der Veer, 2013)
	Client, professionals	Lifeline	+	+	+	+	All	All	Mental health care	Variable	(Bruijnzeels, 2018)
	Client, peers, caregivers	Program for the Education & Enrichment of Relational Skills (PEERS)	+	~	~	+	Adolescence - Young adulthood	≥ Well below average	Mental health care	14 - 16 Sessions	(McVey et al., 2016)
	Client, peers	Self in view	+	+	+	+	School age	≥ Low average	Mental health care	10 Sessions	(Schweizer, 2020)
	Client, peers, caregivers	Socio-Dramatic Affective-Relational Intervention (SDARI)	–	+	~	~	Childhood	≥ Well below average	Mental health care, social domain	Variable	(Lerner et al., 2011)
	Client, partner, family	Systemic Therapy	+	~	+	~	All	All	Mental health care, social domain	Variable	(Hazelrigg et al., 1987)
Meso	Client, employers, schools	WorkWeb Autism	+	–	–	+	Adults	Average - above	Self-help	Variable	(Dorland et al., 2021)
Macro	Society	Autism Experience	~	+	–	+	All	All	Public domain	Variable	(https://www.autismeexperience.nl/, n.d.)

Please note that we realize that Table 2 is not exhaustive. It displays what our program committee currently considers useful at this ‘state of the art’ of this ever evolving and expanding field. We are aware that, as we write this article, innovations are ongoing and other promising programs (e.g., White et al., 2019; Oshima et al., 2022; Shaffer et al., 2023) are upcoming. We therefore primarily give this overview to increase awareness in all stakeholders considering the abundant field of support and services already available. As such, we do not have the intention to advocate for one versus the other program/‘product’, rather we advocate for better disseminating, utilizing, integrating and optimizing the rich available knowledge-base and expertise. When combining methods, providers might deviate from protocolled delivery. Yet, if protocolled delivery is not showing satisfying effects within reasonably expected time, then reasoned deviation based on argumentation relying on knowledge of—and experience with—the working mechanisms of methods should be allowed and encouraged.

## Conclusions, theoretical and practical implications

Integrating insights and reflecting on the results of the previous process (i.e., GoG & DwooA; ‘Coping with neurodivergence’; over the period of 2015–2023), we formulated the following hypothetical conceptualizations, as these may form the groundwork of our crescent in understanding (i.e., the subsequent phases of this iterative development process):

Undergoing events in the outer world continuously alters evaluations of the outer world and impacts experiences in both the outer and inner world, which creates a ‘conceptual crisis’ when outer world experiences do not fully match with internal mental representations and predictions (e.g., Beck, 2011; van de Cruys et al., 2014). Thus, with ‘conceptual crisis’ referring to the fact that conceptual reasoning is an important capacity that can help people get through life as conceptualizations of the world help them to better understand and thus navigate that world. However, when things in the outer world change and therefore a person’s mental representation (conceptualization) of those things needs to change (be adapted/updated), this mental exercise costs mental resources (i.e., energy and headspace). A person will need processing time and dedicated attention to re-consider the concepts they have about their self, about human kind in more general terms and about the larger human and non-human world. Such a conceptual reasoning task is layered (i.e., ranging from conscious to unconscious, concerning automatic thoughts, intermediate and core beliefs) and pulls from (1) rational analytical cognitive resources (i.e., cortical activity, such as language processing/production), (2) emotional (e-motion > experiences/sensations that ‘move’ into motion!)/intuitive cognitive resources (i.e., limbic activity), (3) integrative areas (i.e., medial pre-frontal areas involved in for instance social motivation/cognition), and (4) physical somatic resources (i.e., cerebellum and autonomic nervous system, i.e., sensory input and motor movement!). As such, this is a complex, multi-layered mental as well as somatic activity that is exhausting for any person, but for neurodivergent people in particular, as their mental maturation process (i.e., ripening of several different brain areas and functions) is hypothesized to be progressing at a different order and/or different speed in a potentially differently experienced environment (e.g., Crowell et al., 2009; Silva et al., 2013; van de Cruys et al., 2014; Brown, 2020). Therefore, this exhausting exercise can, on the long run, overwhelm them too much, leading to ‘crisis’, i.e., immobilization, getting stuck in a passive mode, not being able to get started (hypo-arousal), i.e., autistic inertia (Buckle et al., 2021). Alongside, autistic inertia not only entails that it causes this long-term de-activation, but, usually on the shorter term, it can also involve not being able to stop, or even accelerating, and getting in a hyper-active mode (hyper-arousal/hypervigilance). This probably entails over-activity of the stress-system, i.e., the autonomic nervous system and the limbic system, and in particular the amygdala. This phenomenon has also popularly been referred to as the ‘amygdala-hijack.’^1^ Yet, we prefer the more neutral term ‘dysregulation’, as probably the heightened/altered activity of the amygdala, might be combined with—or alternated with—a concurrent over-activity of the cortex, possibly prioritizing cortical information, i.e., ‘over-thinking’ everything too much (e.g., Leonard, 2023). Finally, this processing might also absorb much of the activity of the medial pre-frontal areas, involved in working-memory etc., in that way prioritizing this intermediate area for analytical activity, rather than passing through emotional/physical information to the cortex, i.e., suppressing conscious awareness/experience of emotions, so-called limited interoception or alexithymia (if mainly unconscious) or experiential avoidance (if more subconscious). If this process is so ‘pre-occupying’ (oriented at short term survival rather than realizing longer term needs/goals) people can feel stuck in fight/flight/freeze/fawn ‘mode’ and might temporarily need another person to co-regulate them, yet on their own terms (remaining autonomy and authenticity). Thus, a careful balance is needed, in which near ones need to learn by experience when to activate or when to limit social pressure, as the person needs to be driven by the right mix of intrinsic and extrinsic motivation. Moreover, if broader members from society could interpret certain behaviors as ‘getting stuck’, rather than limited motivation, this would reduce friction and frustrations on both ends, which could allow for more developmental space to get ‘unstuck’.

Please note that above claims are strictly hypothetical. However, in our experience, many neurodivergent people seem to relate to similar ‘body–mind-(dis)connection’ conceptualizations. This could be the case because such explanatory models (often grounded on poly-vagal theory and trauma; e.g. Brown, 2020) are currently gaining popularity (i.e., are applied in several mental health care settings) and have therefore been picked up from their clinicians by our autistic participants, although debunking initiatives of these theories also exist. These ideas would however not have been adopted by these autistic people, if they could not closely relate their experiences to these conceptual ideas. Therefore, we consider these conceptualizations a helpful framework for neurodivergent people to understand their day-to-day experiences and feel supported in these experiences. As such, these conceptualizations can provide a groundwork to build interventions in preventing to get stuck and in getting unstuck. To enable neurodivergent people to better cope with daily life challenges, we thus suggest the importance of learning how to use, dosage, mix and balance these different modes/states. So over the longer term, not just mainly focusing on cognitive knowledge programs, or primarily focusing on emotional experiential programs, but learning how to combine components from both orientations, could be helpful in managing day-to-day life experiences for autistic people. This should be done in a custom-made manner of equally shared decision making, by the person itself, with adequately balanced support of their near ones and professionals involved. Over time, together they learn to better balance and manage this process, based on shared knowledge and skills in regulation of sensory, emotional and cognitive experiences, i.e., the learning experiences they build up together.

To finish off with, we want to emphasize that alongside this clinical stance, we as the AWA consortium are also taking on an empirical stance. Therefore, in the following stages of this shared iterative process, we will further co-create and empirically evaluate the integrative approach, that aims to support identity development by facilitating teachable moments and concrete actions towards self-realization, and by teaching about authenticity and social identity as products of reciprocal trust, craft and tolerance (De Graaff et al., 2023). Any reactions or suggestions for these future shared endeavors are welcomed (k.greaves-lord@rug.nl).

## Data availability statement

The data analyzed in this study is subject to the following licenses/restrictions: these are qualitative transcripts that can be retrieved from the first authors. Requests to access these datasets should be directed to k.greaves-lord@lentis.nl.

## Ethics statement

The studies involving human participants were reviewed and approved by TWOR Maasstadziekenhuis. The patients/participants provided their written informed consent to participate in this study.

## Author contributions

All authors listed have made a substantial, direct, and intellectual contribution to the work and approved it for publication.

## Footnotes

^1^ https://en.wikipedia.org/wiki/Amygdala_hijack

## Supplementary material

Supplementary Figure 1 Depiction of inter-relations between themes derived from GoB and DWooA results.

## References

[ref9009] Agterberg-Rouwhorst S. (2022). Vind je eigen weg met jouw autisme. Huizen: Pica.

[ref9019] Bartholomeus P. J. P. W. (2021). ReAttach: A transdiagnostic intervention for adults and children with mental health problems. Maastricht: Maastricht University.

[ref1] Beck J. S. (ed.) (2011). Cognitive Behavior Therapy: Basics and Beyond 2. Guilford Press A Division of Guilford Publications, Inc. New York, NY.

[ref2] Braun V. Clarke V. (2006). Using thematic analysis in psychology. Qual. Res. Psychol. 3, 77–101. doi: 10.1191/1478088706qp063oa, PMID: 37556436

[ref9015] Breitbart W. S. Poppito S. R. (2014). Individual meaning-centered psychotherapy for patients with advanced cancer. Cary: Oxford University Press, Incorporated. 10.1200/JCO.2011.36.2517 PMC3646315 22370330

[ref9024] Brom D. Stokar Y. Lawi C. Nuriel-Porat V. Ziv Y. Lerner K.. (2017). Somatic Experiencing for posttraumatic stress disorder: A randomized controlled outcome study. Traumatic Stress 30, 304–312. 10.1002/jts.22189 PMC5518443 28585761

[ref3] Bronfenbrenner U. (1979). The Ecology of Human Development. Cambridge, MA: Harvard University Press.

[ref4] Brown D. (2020). Polyvagal theory and regulating our bodily state. Affect Autism

[ref9032] Bruijnzeels M. (2018). Levenslijn opschrijven. Verkregen via https://www.autismevanuitdetail.nl/autisme-bij-vrouwen/levenslijn-opschrijven/

[ref5] Buckle K. L. Leadbitter K. Poliakoff E. Gowen E. (2021). No way out except from external intervention: first-hand accounts of autistic inertia. Front. Psychol. 12:631596. doi: 10.3389/fpsyg.2021.631596, PMID: 34326790 PMC8314008

[ref9017] Buys P. Aerts I. (2011). Mijn zelfportret: Groepswerk rond zelfbeeld voor adolescenten met autisme. Antwerpen: Garant.

[ref9023] Classen C. C. Hughes L. Clark C. Hill-Mohammed B. Woods P. Beckett B. (2021). A pilot RCT of a body-oriented group therapy for complex trauma survivors: An adaptation of Sensorimotor Psychotherapy. Trauma & Dissociation 22, 52–68. 10.1080/15299732.2020.1760173 32419670

[ref6] Coyne I. T. (1997). Sampling in qualitative research. Purposeful and theoretical sampling; merging or clear boundaries? J. Adv. Nurs. 26, 623–630. doi: 10.1046/j.1365-2648.1997.t01-25-00999.x, PMID: 9378886

[ref7] Crowell S. E. Beauchaine T. P. Linehan M. M. (2009). A biosocial developmental model of borderline personality: elaborating and extending Linehan’s theory. Psychol. Bull. 135, 495–510. doi: 10.1037/a0015616, PMID: 19379027 PMC2696274

[ref8] De Graaff B. Alma M. A. Stoffels A. J. Sloot L. Jagersma G. Mandemaker H.. (2023). Identity development in autism. Wetenschappelijk Tijdschrift Autisme 22, 19–34.

[ref9] Dell’Osso L. Amatori G. Massimetti G. Nardi B. Gravina D. Benedetti F.. (2022). Investigating the relationship between autistic traits and symptoms and catatonia Spectrum. Eur. Psychiatry 65:e81. doi: 10.1192/j.eurpsy.2022.2334 36328964 PMC9724219

[ref10] Demetriou E. Lampit A. Quintana D. Naismith S. L. Song Y. J. C. Pye J. E.. (2018). Autism spectrum disorders: a meta-analysis of executive function. Mol. Psychiatry 23, 1198–1204. doi: 10.1038/mp.2017.75, PMID: 28439105 PMC5984099

[ref9037] Dorland H. Landsman J. Miedema H. Brouwer S. (2021). WerkWeb-Autisme: Tool bij de begeleiding van werknemers met autisme. Bedrijfs- en Verzekeringsgeneeskunde 29, 32–35.

[ref9018] Frazão A. Santos S. Lebre P. (2023). Psychomotor intervention practices for children with autism spectrum disorder: A scoping review. Review Journal of Autism and Developmental Disorders 10, 319–336.

[ref9020] Idris S. van Pelt B. J. Jagersma G. Duvekot J. Maras A. van der Ende J.. (2022). A randomized controlled trial to examine the effectiveness of the Dutch version of the Program for the Education and Enrichment of Relational Skills (PEERS®). BMC Psychiatry. 22:293. doi: 10.1186/s12888-022-03913-3 35459118 PMC9034592

[ref11] Greaves-Lord K. Kruizinga I. Landssman J. van Daalen E. Landlust A. Balkom I. D. C. (2022). Factoren rond gedragsproblemen bij ASS. Wetenschappelijk Tijdschrift Autisme 2, 2–25.

[ref12] Guba E. G. (1981). Criteria for assessing the trustworthiness of naturalistic inquiries. ECTJ 29, 75–91.

[ref9001] Hahs A. D. Dixon M. R. Paliliunas D. (2019). Randomized controlled trial of a brief Acceptance and Commitment Training for parents of individuals diagnosed with autism spectrum disorders. Contextual Behavioral Science 12, 154–159.

[ref9005] Hartmann K. Urbano M. Manser K. Okwara L. (2012). “Modified Dialectical Behavior Therapy to improve emotion regulation in autism spectrum disorders” in Autism spectrum disorders. eds. Richardson C. E. Wood R. A. (New York: Nova Science Publishers), 41–72.

[ref9036] Hazelrigg M. D. Cooper H. M. Borduin C. M. (1987). Evaluating the effectiveness of family therapies: An integrative review and analysis. Psychological Bulletin 101, 428–442. 3602249

[ref9003] Hesselmark E. Plenty S. Bejerot S. (2014). Group Cognitive Behavioural Therapy and group recreational activity for adults with autism spectrum disorders: A preliminary randomized controlled trial. Autism 18, 672–683. 24089423 10.1177/1362361313493681 PMC4230566

[ref9013] https://www.autismedigitaal.nl/avn/imaginaire-rescripting/ (n.d.).

[ref9038] https://www.autismeexperience.nl/ (n.d.).

[ref9016] Kaltenegger H. C. Philips B. Wennberg P. (2020). Autistic traits in Mentalization-based Treatment for concurrent borderline personality disorder and substance use disorder: Secondary analyses of a randomized controlled feasibility study. Scandinavian Journal of Psychology 61, 416–422. 31840273 10.1111/sjop.12595

[ref9025] Kraijenhoff L. (2016). Het vollehoofdenboek: Een werkboek voor kinderen en volwassenen. Den Haag: Acco.

[ref9014] Kuipers-Hemken M. Buma S. (2020). Minder stress, minder autisme: Plan C. Amsterdam: SWP.

[ref13] Leonard E. (2023). Three origins of overthinking. Psychol. Today

[ref9035] Lerner M. D. Mikami A. Y. Levine K. (2011). Socio-dramatic affective-relational intervention for adolescents with Asperger syndrome & high functioning autism: Pilot study. Autism 15, 21–42. 20923890 10.1177/1362361309353613

[ref9008] Lobregt-van Buuren E. Sizoo B. Mevissen L. de Jongh A. (2019). Eye Movement Desensitization and Reprocessing (EMDR) therapy as a feasible and potential effective treatment for adults with autism spectrum disorder (ASD) and a history of adverse events. Autism and Developmental Disorders 49, 151–164. 10.1007/s10803-018-3687-6 30047096

[ref14] López B. Leekam S. R. Arts G. R. (2008). How central is central coherence? Preliminary evidence on the link between conceptual and perceptual processing in children with autism. Autism 12, 159–171. doi: 10.1177/1362361307086662, PMID: 18308765

[ref9007] Mazefsky C. A. White S. W. Beck K. B. Conner C. M. (2021). “Emotion Awareness and Skills Enhancement (EASE) program” in Encyclopaedia of autism spectrum disorders. ed. Volkmar F. R. (New York: Springer International Publishing), 1688–1693.

[ref9033] McVey A. J. Dolan B. K. Willar K. S. Pleiss S. Karst J. S. Casnar C. L.. (2016). A replication and extension of the PEERS for young adults social skills intervention: Examining effects on social skills and social anxiety in young adults with autism spectrum disorder. Autism and Developmental Disorders 46, 3739–3754. 10.1007/s10803-016-2911-5 PMC5310211 27628940

[ref9027] Meins I. A. Muijsson-Bouwman D. C. Nijman S. A. Greaves-Lord K. Veling W. Pijnenborg G. H. M.. (2023). VR-SOAP, a modular Virtual Reality Treatment for Improving Social Activities and Participation of young people with psychosis: A study protocol for a single-blind multi-centre randomized controlled trial. Trials 24, 1–11. 37061694 10.1186/s13063-023-07241-z PMC10105944

[ref15] Oshima F. Mandy W. Seto M. Hongo M. Tsuchiyagaito A. Hirano Y.. (2022). Awareness and care for my autistic traits (ACAT) program for adolescents with autism spectrum disorders: a multicenter randomized controlled trial, 26 October 2022, PREPRINT (version 1) available at Research Square. doi: 10.21203/rs.3.rs-2123169/v1

[ref9021] Porges S. W. Bazhenova O. V. Bal E. Carlson N. Sorokin Y. Heilman K. J.. (2014). Reducing auditory hypersensitivities in autistic spectrum disorder: Preliminary findings evaluating the listening project protocol. Frontiers in Pediatrics 2, 1–10. 25136545 10.3389/fped.2014.00080 PMC4117928

[ref16] Sandelowski M. (1993). Rigor or rigor mortis: the problem of rigor in qualitative research revised. Adv. Nurs. Sci. 16, 1–8. doi: 10.1097/00012272-199312000-00002, PMID: 8311428

[ref9034] Schweizer C. (2020). Art therapy for children diagnosed with autism spectrum disorders: Development and first evaluation of a treatment programme. Groningen: University of Groningen.

[ref9029] Semple R. J. (2019). Review: Yoga and mindfulness for youth with autism spectrum disorder: review of the current evidence. Child Adolesc Ment Health. 24, 12–18. doi: 10.1111/camh.12295. Epub 2018 Aug 13, PMID: 32677240

[ref17] Shaffer R. C. Schmitt L. M. Reisinger D. L. Coffman M. Horn P. Goodwin M. S.. (2023). Regulating together: emotion dysregulation group treatment for ASD youth and their caregivers. J. Autism Dev. Disord. 53, 1942–1962. doi: 10.1007/s10803-022-05461-x, PMID: 35141815 PMC10126211

[ref18] Silva E. B. Filipini R. Monteiro C. B. Valenti V. E. de Carvalho S. M. Wajnsztejn R.. (2013). The biopsychosocial processes in autism spectrum disorder. Int. Arch. Med. 6:22. doi: 10.1186/1755-7682-6-22 23656636 PMC3671139

[ref9004] Stella B. Kwakman M. Boyer B. E. (2022). Effectiveness of Competitive Memory Training (COMET) for low self-esteem in youth with autism spectrum disorder: A randomized controlled pilot study. Autism 9, 1–11.

[ref1001] The Hague Health Council of the Netherlands. (2009). Autism spectrum disorders: a lifetime of difference. Publication no. 2009/09.

[ref9002] Vancampfort D. Brunner E. van Damme T. Stubbs B. (2022). Efficacy of basic Body Awareness Therapy on functional outcomes: A systematic review and meta-analysis of randomized controlled trials. Physiotherapy Research International 28, 1–12. 10.1002/pri.1975 36103584

[ref19] van de Cruys S. Evers K. van der Hallen R. van Eylen L. Boets B. de-Wit L.. (2014). Precise minds in uncertain worlds: predictive coding in autism. Psychol. Rev. 121, 649–675. doi: 10.1037/a0037665, PMID: 25347312

[ref20] Van den Bosch K. Greaves-Lord K. (2019). Kennisagenda Autisme. Onderzoek onder stakeholders naar onderzoeksbehoeften. Autisme Magazine 4:36.

[ref21] Van der Linden K. Simons C. Viechtbauer W. Ottenheijm T. Marcelis M. (2021). A momentary assessment study on emotional and biological stress in adult males and females with autism spectrum disorder. Sci. Rep. 11:14160. doi: 10.1038/s41598-021-93159-y 34238944 PMC8266874

[ref9026] Van der Meer L. Jonker T. Wadman H. Wunderink C. van Weeghel J. Pijnenborg G. H. M.. (2021). Targeting personal recovery of people with complex mental health needs: The development of a psychosocial intervention through user-centered design. Frontiers in Psychiatry 12, 1–18. 10.3389/fpsyt.2021.635514 PMC8060492 33897494

[ref9010] Van der Meijden S. van der Stegen B. (2009). ‘Ik heb iets van autisme of zo…’: Psycho-educatie voor slimme jongeren met ASS. Kind & Adolescent Praktijk 4, 187–197.

[ref9031] Van der Veer W. G. (2013). Het effect van de ASSwijzer. Amsterdam: Medix.

[ref9006] Van Pelt B. J. Nijman S. A. van Haren N. E. M. Veling W. Pijnenborg G. H. M. van Balkom I. D. C.. (2022). Dynamic Interactive Social Cognition Training in Virtual Reality (DiSCoVR) for adults with autism spectrum disorder: A feasibility study. Research in Autism Spectrum Disorders 96, 1–13.

[ref9030] Van Wamel A. Planije M. (2018). Implementatie van de Yucelmethode: Een handreiking. Utrecht: Trimbos-instituut.

[ref9028] Veling W. Lestestuiver B. Jongma M. Hoenders H. J. R. van Driel C. (2021). Virtual reality relaxation for patients with a psychiatric disorder: Crossover randomized controlled trial. Medical Internet Research 23, 1–14. 10.2196/17233 PMC7846446 33448933

[ref9012] Vermeulen P. (2005). Ik ben speciaal: Werkboek psycho-educatie voor mensen met autisme. Berchem: Epo.

[ref9011] Visser K. Greaves-Lord K. Dekker L. Boudesteijn F. Maras A. van der Vegt E. (2012). Het Ik Puber onderzoek: Een onderzoek naar de Ik Puber-training, een training ter bevordering van de psychoseksuele en puberteitsontwikkeling bij jongeren met een stoornis in het autismespectrum. Wetenschappelijk Tijdschrift Autisme 11, 132–137.

[ref9022] Vuijk R. Deen M. Geurts H. M. Arntz A. (2023). Schema Therapy for personality disorders in autistic adults: Results of a multiple case series study. Clinical Psychology & Psychotherapy 30, 458–472. 36522138 10.1002/cpp.2817

[ref22] White S. W. Smith I. C. Miyazaki Y. Conner C. M. Elias R. Capriola-Hall N. N. (2019). Improving transition to adulthood for students with autism: a randomized controlled trial of STEPS. J. Clin. Child Adolesc. Psychol. 50, 187–201. doi: 10.1080/15374416.2019.1669157 31609666 PMC8513749

