# Supplementary figures and images for: Clinical stance on response initiation in autistic adults: co-creating an integrative approach based on theory and lived experiences to act from language, via motor movement to wellbeing
Source: Front Psychol. 2023 Sep 14;14:1229596. doi: 10.3389/fpsyg.2023.1229596 (PMC10539615; doi:10.3389/fpsyg.2023.1229596)

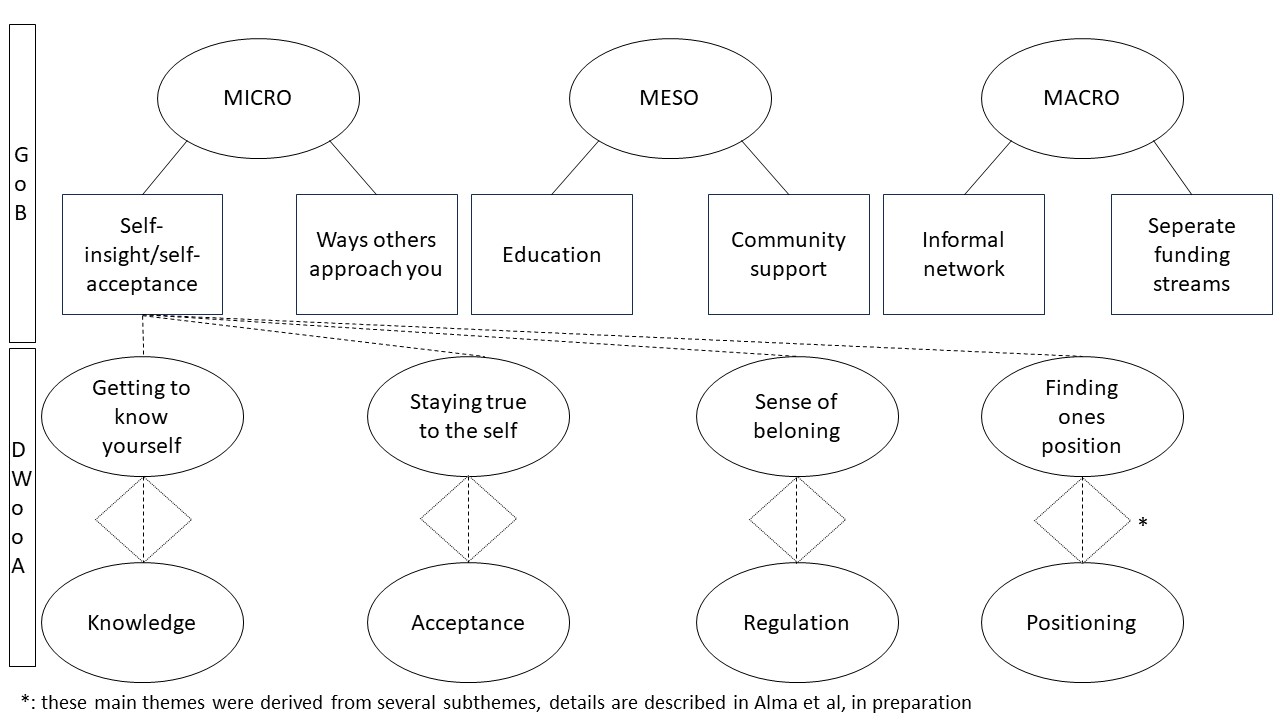

Supplement: Supplementary Figure 1 — Depiction of inter-relations between themes derived from GoB and DWooA results. [file Image_1.jpg]
